# Surface Antigen 1 Is a Crucial Secreted Protein That Mediates *Babesia microti* Invasion Into Host Cells

**DOI:** 10.3389/fmicb.2019.03046

**Published:** 2020-01-15

**Authors:** Muxiao Li, Yangsiqi Ao, Jiaying Guo, Zheng Nie, Qin Liu, Long Yu, Xiaoying Luo, Xueyan Zhan, Yangnan Zhao, Sen Wang, Xiaomeng An, Lan He, Junlong Zhao

**Affiliations:** ^1^State Key Laboratory of Agricultural Microbiology, College of Veterinary Medicine, Huazhong Agricultural University, Wuhan, China; ^2^Key Laboratory of Preventive Veterinary Medicine in Hubei Province, College of Animal Science and Technology, Huazhong Agricultural University, Wuhan, China; ^3^Key Laboratory of Animal Epidemical Disease and Infectious Zoonoses, Ministry of Agriculture, Huazhong Agricultural University, Wuhan, China

**Keywords:** *Babesia microti*, surface antigen 1, secreted protein, *in vitro* culture, invasion

## Abstract

*Babesia microti*, a tick-borne intraerythrocytic zoonotic protozoan, causes most of human babesiosis in the world, and patients usually experience intermittent fever, fatigue, and chills, followed by a combination of additional symptoms and even death in severe cases. Unfortunately, there is no curable drug or effective vaccine available, and the mechanism of related virulence factors in invasion to host cells during the merozoite stage is unclear. Here, we evaluated a secreted protein annotated as *B. microti* surface antigen 1 (BmSA1) and identified from *in vitro* culture supernatant by liquid chromatography coupled with tandem mass spectrometry (LC-MS/MS). BmSA1 fragment was expressed in *Escherichia coli* to prepare polyclonal antiserum. Western blot analysis revealed the existence of BmSA1 in the lysate of the parasites and the hemolysate of infected red blood cells (iRBCs). Laser confocal microscopy confirmed BmSA1 as a secreted protein with diffuse distribution around the parasites in red blood cells (RBCs). The adhesion capacity of BmSA1 against the host RBCs was tested by RBC binding assays using the recombinant BmSA1 protein (rBmSA1), which was shown to specifically bind to host RBCs. Further *in vitro* antiserum-neutralization test demonstrated that the growth of parasites could be significantly inhibited by the anti-BmSA1 antiserum. These results indicate that BmSA1 is a crucial factor for *B. microti* invasion into host RBCs with an important role in host-parasite interactions during the merozoite stage and has the potential use as a vaccine candidate due to its high secretion amount.

## Introduction

*Babesia microti*, an important tick-borne intraerythrocytic protozoan, belongs to the phylum apicomplexa and is one of the several most common human babesiosis pathogens in the world (Uilenberg, 2006; Vannier and Krause, 2012). In nature, *B. microti* is transmitted by *Ixodes* ticks in most cases, and the reported hosts are the white-footed mouse (*Peromyscus leucopus*) and human (Vannier et al., 2015; Krause, 2019).

Human infection with *B. microti* is often asymptomatic, but immunodeficiency patients (such as splenectomy and AIDS patients) and people with poor immunity can be seriously infected, coupled with the symptoms of severe anemia, renal failure, and respiratory distress (Hunfeld et al., 2008). However, there is no gold standard diagnostic in the clinic. Blood donors with asymptomatic infections often do not know their infection, thus posing a significant safety hazard to subsequent blood use in patients (Fang and McCullough, 2016). Since the report of the first case of human babesiosis in Yugoslavia in 1957 (Skrabalo and Deanovic, 1957), thousands of cases have been reported around the world, with an upward trend year by year (Westblade et al., 2017). In recent years, the number of human babesiosis cases reached 2,000 per year in the United States (Krause, 2019), and *B. microti* began to attract worldwide attention due to its widespread distribution in endemic areas, its increased risk for humans and its potential risk in blood transfusion.

*Babesia microti* has a life cycle of two major stages: a sexual stage in ticks and an asexual intraerythrocytic stage in mammalian erythrocytes. At the asexual stage, *B. microti* reproduces by schizogamy, giving rise to a large number of merozoites in the red blood cells (RBCs), causing cell rupture and damage to the host’s circulatory system (Vannier et al., 2015). In this process, *Babesia* will secrete antigens which can help parasites effectively recognize and adhere to host RBCs, then the parasite will form the tight junction between erythrocyte surface and apical part and start invasion (Yokoyama et al., 2006). Due to their direct exposure to the host’s immune system, these antigens are also very effective in stimulating the host’s immune system, causing a host of immune responses, including the humoral and cellular immune responses (Nathaly Wieser et al., 2019). For this reason, the secreted antigen has become a vaccine candidate in developing the *Babesia* vaccine, and it may also facilitate the development of a diagnostic test for babesiosis.

*Babesia microti* surface antigen 1 (BmSA1)has been reported as a diagnostic marker with high reactivity (Cornillot et al., 2016), and the ELISA detection method has also been established (Luo et al., 2011; Thekkiniath et al., 2018). However, there is no relevant report on its function or its specific role in parasite invasion. Therefore, it is necessary to ascertain the biological significance of these secreted proteins.

The purpose of the present study was to identify the localization of *B. microti* SA1 in parasites and its function in the invasion stage. This study will add insight into the invasion of *B. microti* into host RBCs and how secreted proteins help parasites during the merozoite stage.

## Materials and Methods

### Ethics Statement

The experimental animals were housed and treated in accordance with the stipulated rules for the Regulation of the Administration of Affairs Concerning Experimental Animals of China. All experiments were performed under the approval of the Laboratory Animals Research Centre of Hubei Province and Huazhong Agricultural University (Permit number: HZAUMO-2017-040).

### Experimental Animals and Parasite Strain

*Babesia microti* strain ATCC^®^ PRA-99TM was obtained from the Shanghai Veterinary Research Institute (Chinese Academy of Agricultural Sciences, Shanghai, China) and maintained in our laboratory (State Key Laboratory of Agricultural Microbiology, College of Veterinary Medicine, Huazhong Agricultural University, Wuhan, Hubei, China) by serial passage in Kunming mice erythrocytes. Briefly, 10^7^ *B. microti*-infected erythrocytes were injected intraperitoneally into Kunming mice, and parasites were isolated from 80 to 90% infected RBCs. The parasitemia was determined by Giemsa staining of thin blood smears.

### Collection of Red Blood Cells

Red blood cells were collected from mice into tubes containing K_2_.EDTA solution (solution/RBCs = 1:9; 10% K_2_.EDTA) and were centrifuged to pellet the cells (1500 rpm for 10–20 min at room temperature). After removing plasma and buffy layer, the cell pellets were washed in an approximately five-fold RBC volume of PSG solution (Puck’s Saline Glucose solution) configured with 0.016 g CaCl_2_._2_H_2_O, 8.0 g NaCl, 0.4 g KCl, 0.29 g Na_2_HPO_4_.7H_2_O, 0.15 g KH_2_PO_4_, 1.10 g D-glucose, 0.15 g MgSO_4_.7H_2_O, 0.005 g Phenol red and 1.0-liter ddH_2_0. The pH of the solution was adjusted to 7.2 with HCl, filtered through 0.22 μm sterilized membrane and stored at 4°C for further use. All these reagents were purchased from Sigma-Aldrich, St. Louis, MO, United States). After centrifugation at 1500 rpm for 15 min and three washes in the same solution, the supernatant and buffy layer were removed, and the RBC pellets were resuspended in the same volume of PSG + G solution (plus extra 20 g/L glucose solution), 1% Antibiotic/Antimycotic (Corning, Shanghai, China), mixed and passed through a sterile 0.22 μm filter) and stored at 4°C for further use.

### Short-Term *in vitro* Culture

Blood collected from *B. microti*-infected mice was used in a short-term *in vitro* culture as follows. Briefly, 25 μL of infected mouse RBCs (iRBCs) (15% parasitemia) was mixed with 15 μL of uninfected normal RBCs (10% hematocrit) and 110 μL of culture medium supplemented with 2% HB-101 (Irvine Scientific, Shanghai, China), 20% Fetal bovine serum (FBS, ATLANTA Biologicals, Shanghai, China), 10 mg/L Albumax I (Gibco Life Technologies, Shanghai, China), 2 mM L-glutamine (Irvine Scientific, Shanghai, China), 2% Antibiotic/Antimycotic 100× (Corning, Shanghai, China), and hypoxanthine (200 μM)-thymidine (30 μM) (Sigma-Aldrich, St. Louis, MO, United States) in a 96-well plate. The parasites were cultured at 37°C in a microaerophilous stationary phase incubator containing 2% O_2_, 3% CO_2_, and 93% N_2_. Parasitemia was monitored every 24 h by counting a minimum of 2000 RBCs of Giemsa-stained thin-blood smears.

### Liquid Chromatography Coupled With Tandem Mass Spectrometry Analysis of *in vitro* Culture Supernatant

Serum-free medium was used to culture *B. microti in vitro*, and short-term *in vitro* culture was performed as described above. Meanwhile, 100 μL of culture supernatant was collected separately at 4, 8, 18, 24, 48, and 72 h. The samples were denatured for SDS-PAGE and the protein in gels was visualized using the rapid silver staining kit (Beyotime Biotechnology, Shanghai, China) and then assessed by liquid chromatography coupled with tandem mass spectrometry (LC-MS/MS) (PTM BioLab, Hangzhou, China).

### Cloning and Expression of Recombinant BmSA1

The BmSA1 full-length gene was amplified from *B. microti* gDNA by PCR using the specific primers (the sense primer 5′-ATC TAT TCA CTT CTT GCC TGA ACT G-3′and the antisense primer 5′- ATG CCT CCT AAC TGT CAA CTC CG -3′). The specific primers were designed based on the genome sequence of *B. microti* (Silva et al., 2016). The thermal cycling parameters included the activation of Taq polymerase at 95°C for 2 min, 35 cycles of denaturation at 95°C for 20 s, annealing at 56°C for 20 s, extension at 72°C for 25 s, and a final extension of 5 min at 72°C. Then the full-length gene was cloned into the vector pEASY-Blunt (TransGen Biotech, Beijing, China). The truncated fragment (Signal peptide sequence was truncated, amplicon size = 912 bp) of BmSA1 gene were amplified from previously constructed vector using specific primers with homology arms (the sense primer 5′-TTC TGT TCC AGG GGC CCC TGG CTG GTG GTA GTG GTG GTA ATG G-3′ and the antisense primer 5′- ATC GTC AGT CAG TCA CGA TGT TAG AAT AGA AAC ATA GCG ACC GAG G -3′, with homology arms underlined). Amplification was performed in a 50 μL of PCR buffer containing 2.5 U of *TransStart*^®^ *FastPfu* Fly DNA polymerase (TransGen Biotech, Beijing, China), 0.2 μM of each primer, 0.2 mM of each deoxynucleoside triphosphate, 10 μL 5 × *TransStart*^®^ *FastPfu* Fly buffer, and 1 μL of DNA template. PCR was performed at 95°C for 2 min, 35 cycles of 95°C for 20 s, 56°C for 20 s, 72°C for 25 s, and a final extension at 72°C for 5 min.

As the full-length BmSA1 was poorly expressed in *Escherichia coli*, the truncated fragment were amplified and cloned into the expression vector pGEX-6p-1. The recombinant plasmids (pGEX-6p-1-BmSA1) were transformed into *E. coli* BL21 (DE3) strain. The small-scale culture was subjected to 1 mM isopropyl-β-D-thiogalactopyranoside (IPTG) induction for 3 h at 37°C to recombinantly express the BmSA1 protein and evaluate its integrity by SDS-PAGE. BmSA1 was expressed as soluble GST-fusion proteins, and purified using Glutathione Sepharose 4FF resin (General Electric Company, United States) according to the manufacturer’s instructions.

### Preparation of Mouse Anti-BmSA1 Sera

Female Kunming mice (6–8 weeks old) were purchased from the Laboratory Animals Research Centre of Huazhong Agricultural University. Three mice were immunized subcutaneously with 100 μg of purified recombinant BmSA1 (rBmSA1) in an equal volume of Freund’s complete adjuvant (Sigma-Aldrich, St. Louis, MO, United States) for the first injection. The same antigen administration in Freund’s incomplete adjuvant (Sigma-Aldrich, St. Louis, MO, United States) was conducted on day 7, 14, 28, and 32. The anti-BmSA1 sera were collected 14 days after the last immunization while the pre-immune sera were collected before the first immunization and stored at −20°C until further use. The positive serum was collected at 35 days post infection of *B. microti* which were stored at −20°C for further analysis.

### Secretion Assay

Secretion assay was performed as previously described (Carruthers et al., 1999). Briefly, 10^8^ parasitized RBCs were harvested by centrifugation at 1500 rpm for 20 min, followed by removing plasma and buffy layer, adding 500 μL PBS, centrifugation at 1500 rpm for 10 min and two washes of the pellets. Next, the pellets were incubated at 37°C in a water bath with 100 μL of complete medium described above for 2 min before cooling on ice. Secreted supernatants were recovered by three centrifugations of parasites (1000 *g*, 3 min, 4°C; supernatants were transferred to new wells), and the samples were separately treated. The secreted supernatants were compared with a standard amount of lysate from parasites in the following Western blot analysis. The lysate standards of parasites were prepared by adding 0.5 vol of 5 × SDS-PAGE sample buffer to lysates at the same density used for secreted supernatants.

### SDS-PAGE and Western Blot Analysis

After rBmSA1 was subjected to SDS-PAGE electrophoresis, the protein was transferred to the PVDF membranes, followed by blocking the membranes with 0.05% Tween-20 in tris buffered saline (TBST) plus 5% skimmed milk overnight. Next, the membranes were probed separately with the serum from *B. microti*-infected Kunming mice (1:400) and uninfected Kunming mice. The secondary antibody (1:4000) was anti-mouse-IgG-HRP (Bioss, Beijing, China). To identify the native BmSA1, the lysates of *B. microti*-infected RBCs and normal RBCs were analyzed by Western blot using the same procedures as described above. After the samples were blotted on a PVDF membrane, the membrane was probed separately with the anti-BmSA1 antiserum (1:400) and negative mouse serum, using the same secondary antibody (1:4000) as described above. Finally, BmSA1 protein was detected by visualizing the reacted bands using the electrogenerated chemiluminescence (ECL) method.

### Indirect Immunofluorescence Assay (IFA) and Confocal Laser Microscopic Observation

Blood smears were prepared on slides, air-dried and fixed with methanol for 1 min at room temperature. Then, the slides were blocked with PBS (pH 7.2) plus 10% FBS overnight. After three washes with PBS, indirect immunofluorescence staining was performed, using anti-BmSA1 antiserum as the primary antibody and Alexa Fluor 594-conjugated goat anti-mouse IgG (Life Technologies, Inc., Rockville, MD, United States) as the secondary antibody, and nucleus was stained with Hoechst (Beyotime Biotechnology, Shanghai, China). Next, the samples were visualized first under a confocal microscope (Olympus Life Science, Tokyo, Japan) from a normal perspective and then under a confocal laser microscope (Olympus Life Science, Tokyo, Japan) from a three-dimensional perspective. Image analysis was performed using an Axiovision software package (Carl Zeiss Jena, Oberkochen, Germany).

### RBC Binding Assays and RBC Binding Inhibition Assays

Briefly, 100 μL mouse RBCs was washed three times with 500 μL PBS, and then incubated under rotation for 1 h at room temperature with 900 μL of 2.0, 1.0, 0.5, and 0.25 mg/mL of recombinant protein dissolved in PBS. Next, RBCs were collected after centrifugation for 3 min at 1800 *g* and then deposited on 200 μL silicon oil in 1.5 mL centrifuge tubes. After centrifugation for 5 min at 4000 *g*, the proteins attached to the RBCs were recovered in 0.5 mol/L NaCl solution and detected by Western blot using anti-GST Tag McAb.

When performing RBC binding inhibition assays, after mixing 100 μL mouse RBCs washed with PBS with 100 μg/mL rBmSA1, they were incubated with 50, 100, 250, and 500 μg/mL purified anti-BmSA1 antibody, respectively. Then the same steps will be performed and detected by Western blot using anti-GST Tag McAb.

### Antiserum Neutralization Assays

The antiserum neutralization assays were performed by using *in vitro* cultured *B. microti*. The iRBCs were incubated separately with mouse anti-BmSA1 antiserum, pre-immune serum (negative control) or alone with nothing added (blank control) at different concentrations for 2 h at room temperature in 1.5 mL centrifuge tubes, and then cultured at 37°C for 72 h in a 96-well plate. Giemsa-stained thin blood films from each well were prepared, and the percentage of *B. microti*-infected RBCs was determined by counting approximately 2,000 cells using microscopy. The experiment was repeated three times. The percentage for inhibition of parasite invasion (inhibition rate) was determined using the following formula: [1 – percent parasitemia with test sera/percent blank (without sera) parasitemia]/100%.

### Statistical Analysis

All data were analyzed by using GraphPad Prism 6. Error bars represent standard deviations. One-way ANOVA was used to analyze the antiserum neutralization assay results. *P*-value < 0.05 and < 0.01 indicated statistically significant and extremely significant, respectively.

## Results

### Identification of BmSA1 in the *in vitro* Culture Supernatant of *B. microti* by LC-MS/MS

**FIGURE 1:**
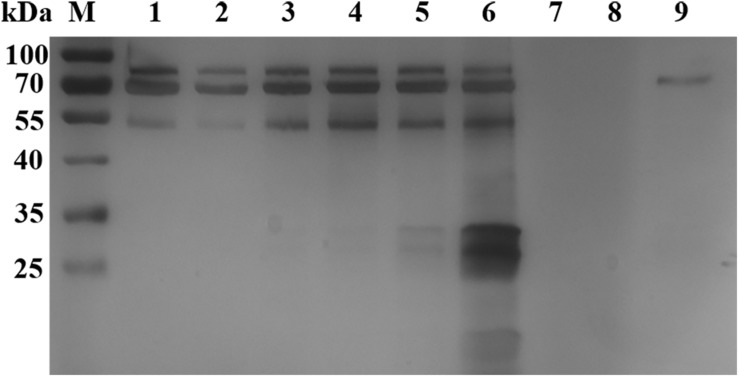
Silver staining analysis of *B. microti in vitro* culture supernatant. Lane M: protein marker; Lanes 1–6: the supernatant samples were cultured *in vitro* for 4, 8, 18, 24, 48, and 72 h, respectively; Lanes 7–8, loading buffer; Lane 9, negative control samples collected after 72 h of incubation.

Silver staining analysis of *B. microti* culture supernatant revealed different bands at 80 and 55 kDa after 4 h of cultivation, and 40–25 kDa bands after 48 h of incubation (Figure 1). A total of 40 proteins were identified from the supernatant, with 20 of them as uncharacterized proteins and 20 as annotated proteins (Supplementary Table S1, LC-MS/MS analysis of *B. microti* culture supernatant). Among the 20 annotated proteins, 03g00785 (BmSA1) was shown by sequence alignment to have a high similarity with the previously reported BMN1-9 gene (data incomplete) (Lodes et al., 2000). Interestingly, BMN1-9 was a secreted antigen screened by serological methods, but its function had not been identified due to technical limitations at that time. So, we selected BmSA1 for further analysis. Bioinformatics analysis found that the BmSA1 has a signal peptide at the N-terminus and its C-terminus has a very specific GPI-anchored modification site. This was basically consistent with the structural features of the GPI proteins in Apicomplexa protozoans. Additionally, BmSA1 has no cysteine rich regions or other specific domains, and this molecular characteristic is similar to that of BmGPI9 and BmGPI10 (two previously reported proteins that can be used as diagnostic markers).

### Cloning and Truncated Expression of BmSA1

**FIGURE 2:**
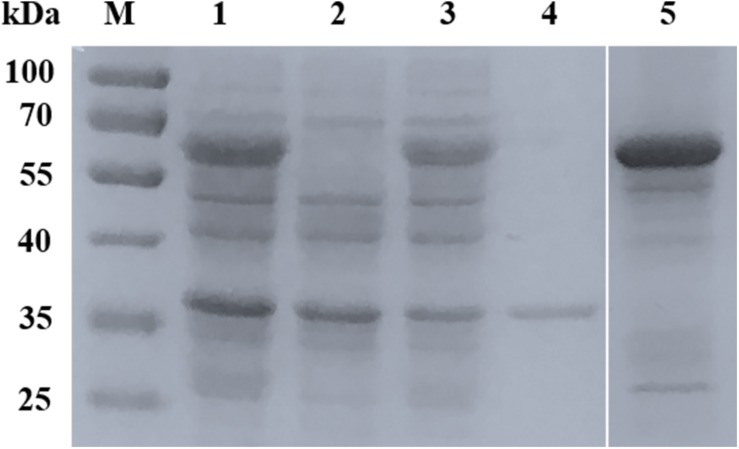
Expression and purification of rBmSA1. SDS-PAGE analysis of the expression of rBmSA1 in *E. coli* BL21 (DE3). Lane M: protein marker; Lane 1: lysate of induced bacterial cells transformed with pGEX-6p-1-BmSA1; Lane 2: lysate of un-induced bacterial cells transformed with pGEX-6p-1-BmSA1; Lane 3: supernatant from lysate of bacterial cells induced by IPTG; Lane 4: pellets from lysate of bacterial cells induced by IPTG; Lane 5: purified rBmSA1 after dialysis and concentration.

The full-length BmSA1 gene was predicted to encode 328 amino acid residues. Due to its poor expression in *E. coli*, BmSA1 without the signal peptide (expressing an expected peptide of ∼33 kDa) was truncated and expressed in pGEX-6p-1 as a soluble 59 kDa GST-fusion protein (Figure 2). The rBmSA1-GST had the same molecular weight as expected and was purified by using Glutathione Sepharose 4FF resin.

### Identification of Anti-BmSA1 Antiserum and Native BmSA1

**FIGURE 3:**
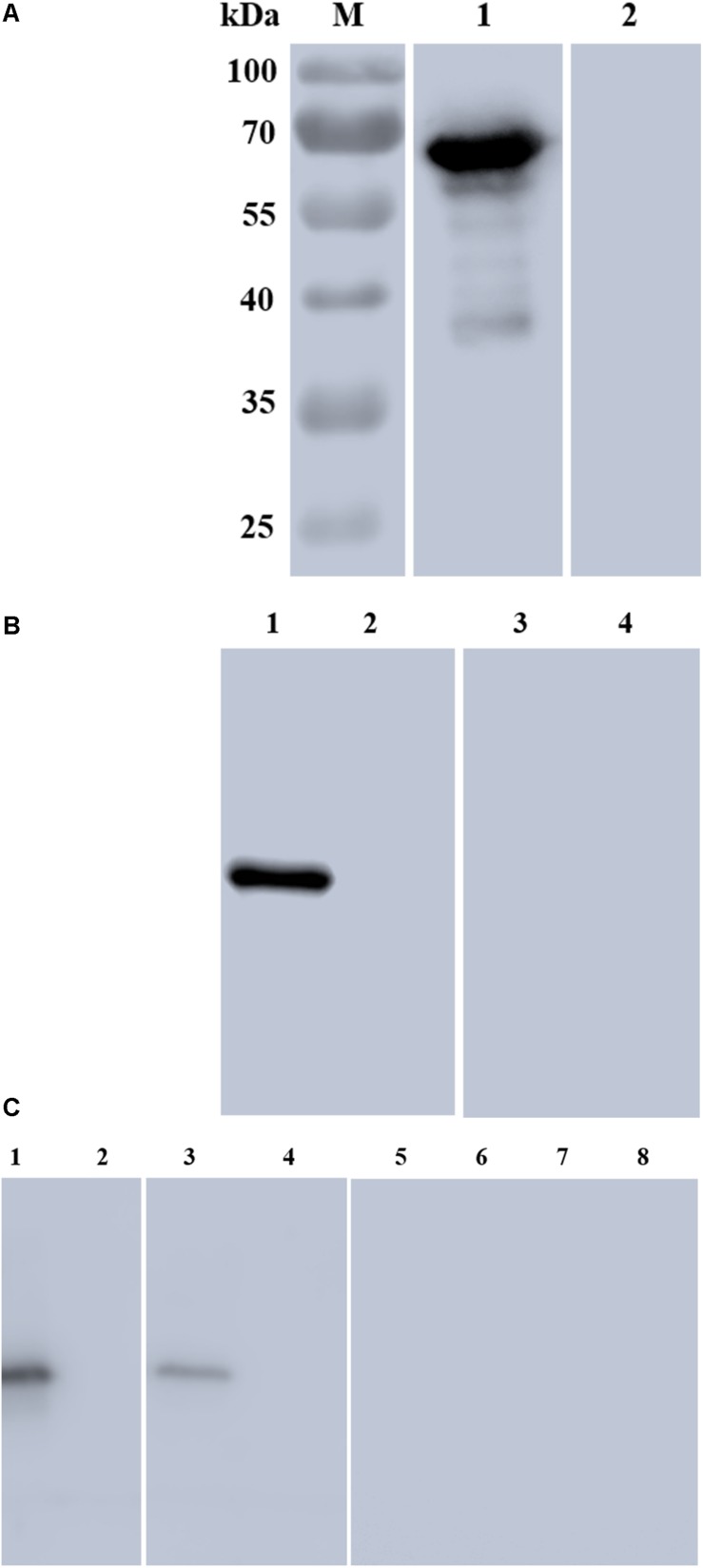
Characterization of rBmSA1 and detection of native BmSA1. **(A)** Lane M: protein marker; Lane 1: rBmSA1 probed with the serum from *B. microti*-infected mice; Lane 2: rBmSA1 probed with the serum from normal mice (negative control); **(B)** Lane 1: lysate of parasites probed with polyclonal anti-BmSA1 antiserum; Lane 2: normal mice RBC lysate probed with polyclonal anti-BmSA1 antiserum; Lanes 3–4: the samples corresponding to 1–2 and probed with the pre-immune serum; **(C)** Lane 1: parasite lysate standards probed with anti-BmSA1 antiserum; Lane 2: normal RBC lysate probed with anti-BmSA1 antiserum; Lane 3: parasite secretion supernatant probed with anti-BmSA1 antiserum; Lane 4: normal RBC supernatant probed with anti-BmSA1 antiserum; Lanes 5–8: samples corresponding to 1–4 and probed with the pre-immune serum.

The rBmSA1-GST expressed by the BmSA1 fragment was recognized by the polyclonal anti-BmSA1 antiserum from mice immunized with the purified recombinant protein, but not by the pre-immune serum. It was also recognized by the serum from *B. microti*-infected mice, but not by the serum from mice uninfected with *B. microti* (Figure 3A). The polyclonal anti-BmSA1 antiserum could specifically recognize the ∼39 kDa full-length BmSA1 protein in *B. microti* lysate, but not normal RBCs and the *B. microti* lysate could not be recognized by pre-immune serum (Figure 3B).

### BmSA1 Is Detectable in the *in vitro* Supernatant

Secretion assay was performed to test whether *B. microti* secretes BmSA1 protein. For this test, 10^8^ parasitized RBCs were harvested to collect their secreted supernatant, and parasite lysate standards were prepared for comparison with the secreted supernatant. Western blot was performed using the polyclonal anti-BmSA1 antiserum, and a single band was identified in both secreted supernatant and the parasite lysate (Figure 3C), indicating the existence of BmSA1 outside *B. microti*. Therefore, BmSA1 was confirmed to be secreted outside the parasites by *B. microti*.

### Subcellular Localization of BmSA1

**FIGURE 4:**
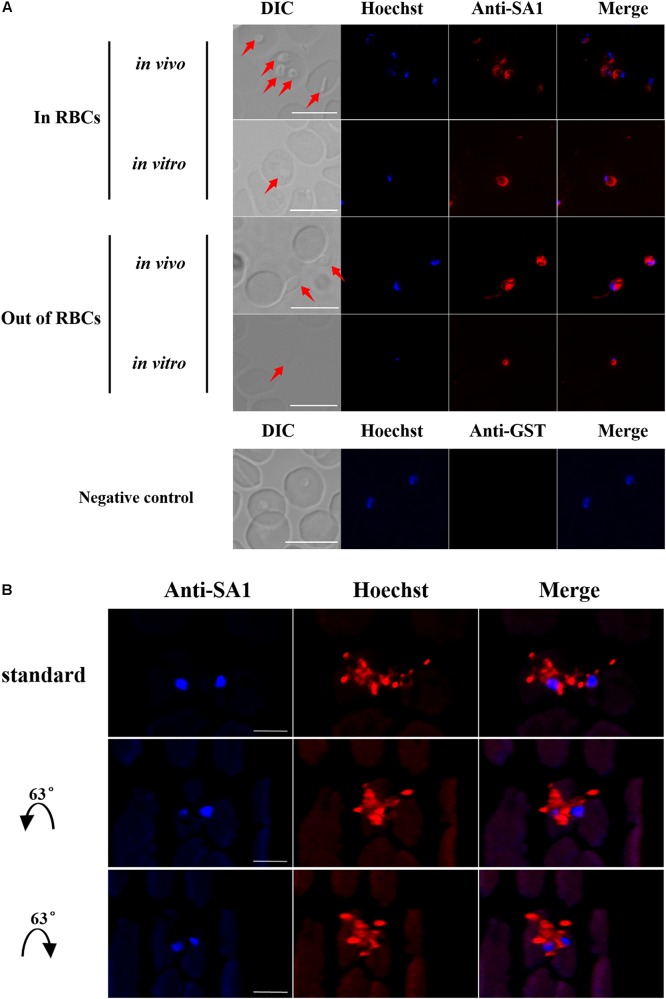
Expression and localization of BmSA1 in *B. microti*. **(A)** IFA indicated that BmSA1 was expressed in different environments (*in vivo* and *in vitro*). BmSA1 was mainly localized in the membrane, with a small amount being distributed in the cytoplasm during the merozoite stage, and it was more expressed when parasites were outside RBCs. The parasite in DIC is indicated by red arrow. Scale bars = 5 μm. **(B)** A three-dimensional perspective of the secretion of BmSA1 by parasites under a laser scanning confocal microscope.

Blood smears were prepared using fresh iRBCs collected separately from mice and *in vitro* cultured iRBCs. BmSA1 was shown to be localized on the cytoplasm and membrane of parasites with a high fluorescence intensity *in vivo* (Figure 4A), indicating that the expression of BmSA1 was abundant. The subcellular localization of BmSA1 in *B. microti* was detected by laser scanning confocal microscopy (LSCM), and BmSA1 was unveiled to be diffusely distributed around the parasites, implying that BmSA1 may exist as a secreted protein (Figure 4B).

### rBmSA1 Can Adhere to Mouse RBCs

**FIGURE 5:**
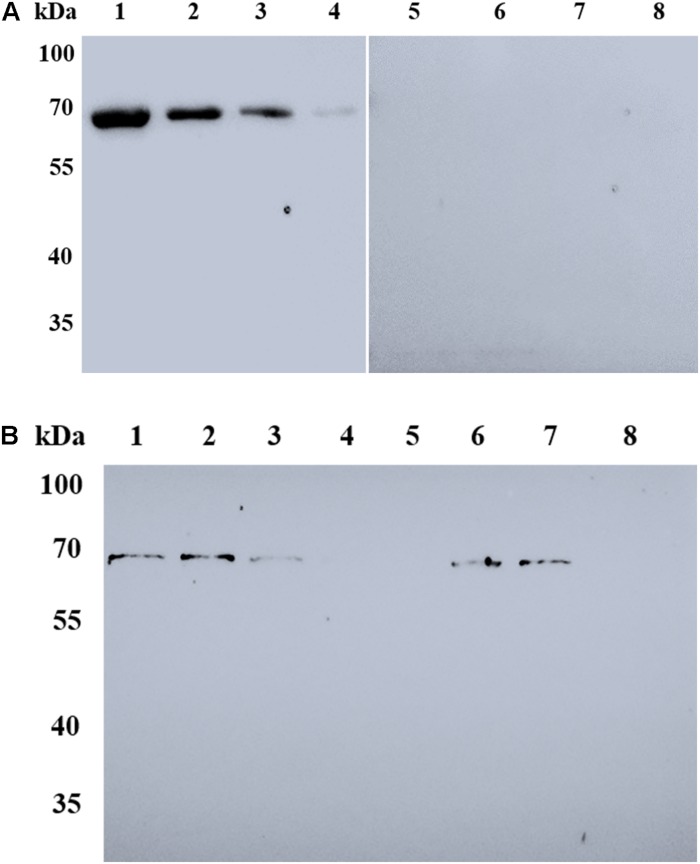
Western blot analysis of RBC binding to rBmSA1. **(A)** Lanes 1–4: mouse RBC binding to rBmSA1 at concentrations of 2.0, 1.0, 0.5, and 0.25 mg/mL, respectively; Lanes 5–8: mouse RBC binding to GST (Negative control) with the same concentrations of rBmSA1. All lanes were reacted with anti-GST antibodies. **(B)** Lane 1: mouse RBC binding to rBmSA1 at concentrations of 0.1 mg/mL; Lanes 2–5: mouse RBC binding to rBmSA1 at concentrations of 0.1 mg/mL with 50, 100, 250, and 500 μg/mL purified anti-BmSA1 antibody, respectively; Lane 6: mouse RBC binding to rBmSA1 at concentrations of 0.1 mg/mL with 500 μg/mL purified mouse pre-immune serum; Lane 7: mouse RBC binding to rBmSA1 at concentrations of 0.1 mg/mL with 500 μg/mL purified anti-GST antibody; Lane 8: mouse RBC.

In order to determine whether BmSA1 binds to host RBCs and plays a role in *B. microti* invasion of RBCs, we investigated whether the protein can adhere to host RBCs by Western blot analysis of purified rBmSA1 protein separately at the concentrations of 2.0, 1.0, 0.5, and 0.25 mg/ml, using GST protein as a control. The RBC binding assay performed with rBmSA1 revealed that rBmSA1 could adhere to mouse RBCs, and the adhesion intensity varied with the concentration of rBmSA1, with the strongest adhesion was observed in 2.0 mg/ml rBmSA1 and no signal was detected in the GST control (Figure 5A). RBC binding inhibition assays showed purified anti-BmSA1 antibody could inhibit rBmSA1 adhesion to RBCs, it revealed the adhesion between rBmSA1 protein and mouse RBC was specific (Figure 5B).

### Anti-BmSA1 Antiserum Inhibits Merozoite Invasion

**FIGURE 6:**
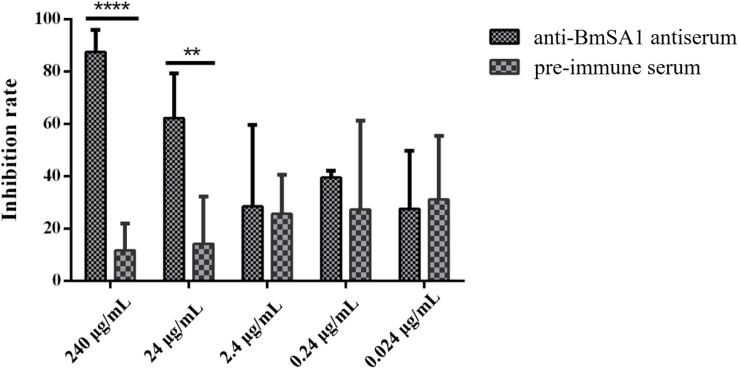
Anti-BmSA1 antiserum neutralization test. Polyclonal anti-BmSA1 antiserum and a negative control (pre-immune serum) were added to *B. microti in vitro* culture. The differences between anti-BmSA1 and pre-immune serum in the relative percentages of invasion were examined by the One-way ANOVA, and *P* values are indicated (^∗^*P* < 0.05, ^∗∗^*P* < 0.01, ^∗∗∗^*P* < 0.001, ^∗∗∗∗^*P* < 0.0001).

In the antibody neutralization assays, 240, 24, 2.4, 0.24, and 0.024 μg/mL antisera were separately used to treat the *in vitro* cultured *B. microti*, followed by counting the parasitemia in 2000 × RBCs and calculating the inhibition rate. The results indicated that the mean inhibition rate of 240, 24, 2.4, 0.24, and 0.024 μg/mL antisera was 87.4 ± 6.99, 62.25 ± 13.92, 28.45 ± 25.42, 39.48 ± 2.14, and 27.51 ± 18.12, respectively. Meanwhile, in the pre-immune serum treated group with the same concentrations, the mean inhibition rate was 11.66 ± 8.43, 14.18 ± 14.69, 25.61 ± 12.26, 27.31 ± 27.70, and 31.11 ± 19.89, respectively (Figure 6). These data demonstrated that anti-BmSA1 antiserum can significantly inhibit the growth of parasites at a concentration of 240 μg/mL (*P* < 0.0001) and 24 μg/mL (*P* < 0.01) after 72 h of cultivation. There was no leukocyte present in *in vitro* cultivation and the effect of anti-BmSA1 antiserum on parasites was limited, probably because of the specificity of the antiserum on inhibiting the growth of parasites rather than killing them.

## Discussion

Lodes et al. (2000) screened out the secreted antigens of *B. microti* MN1 strain by serologic tests in 2000, then Homer et al. (2003) identified several other secreted antigens by the same method and verified their antigenicity in 2003, but the functions of these antigens remained to be elucidated. Due to the limited technology at that time, the *in vitro* culture of *B. microti* had not been established and thus no further research was performed for the secreted antigens. In the present study, we collected the culture supernatant from continuous *in vitro* culture within a short time and identified 40 proteins by LC-MS/MS analysis, including 20 hypothetical proteins, such as 03g4550. GPI prediction (PreGPI)^1^ and signal peptide prediction (SignalP)^2^ were carried out for assessing whether these hypothetical proteins were secreted antigens. Amino acid sequence alignment analysis revealed that 03g04550 was similar to the secreted antigens from *Plasmodium falciparum* and *Babesia bovis* (unpublished data). This result was consistent with previous reports from Elton et al. (2019).

Of the 20 annotated proteins, one protein is named BmSA1 (03g00785), which was a homologous gene of BMN1-9 (Lodes et al., 2000). BMN1-9 was an antigen with excellent antigenicity and immunogenicity as well as a signal peptide sequence at the N-terminus. Interestingly, this protein only exists in the *B. microti* MN1 strain and our parasite is PRA99 strain, with a high homology with R1 strain. These amino acid sequences were examined by maximum likelihood analysis using a phylogenetic tree, and BMN1-9 and BmSA1 were found to be homologous genes (Cornillot et al., 2016). Due to the incomplete genome of BMN1-9, we obtained the full-length BmSA1 gene from *B. microti* gDNA using the primers designed from BmSA1. BmGPI proteins had a similar structure to the merozoite surface antigen family in the protozoan parasite of the phylum Apicomplexa, such as merozoite surface antigens (MSAs) in *B. bovis* (Florin-Christensen et al., 2002; Mosqueda et al., 2002), surface antigen proteins (SAGs) in *Toxoplasma gondii* (Grimwood and Smith, 1992, 1996; Lekutis et al., 2000), and merozoite surface proteins (MSPs) in *P. falciparum* (Gerold et al., 1996; Low et al., 2007; Cowman et al., 2017). During the first step in the erythrocyte invasion of parasites, *Babesia* and *Plasmodium* species use these surface antigens located on the parasite surface coat to bind the host cells (Yokoyama et al., 2006), and these molecules can also be secreted out of the parasites at the same time.

In an effort to understand the role of BmSA1, we expressed the BmSA1 fragment (without signal peptide) and constructed a 59 kDa recombinant protein of BmSA1 (rBmSA1-GST) with GST-tag. The responses of the serum from *B. microti*-infected mice to rBmSA1-GST were detected by Western blot analysis. The results showed that rBmSA1 has a good immunoactivity. BmSA1 was also identified to have high immunogenicity by Western blot analysis of *B. microti* crude protein with the polyclonal anti-BmSA1 antiserum. Both LC-MS/MS and secretion assays showed the presence of BmSA1 in the *in vitro* supernatant, indicating that BmSA1 exists in *B. microti* in two forms. This characteristic was consistent with that of most merozoite surface coat proteins in apicomplexan parasites (Man et al., 2017; Thekkiniath et al., 2018). One of the two existing forms is focused on the GPI-attachment site, where the proprotein cleavage occurs and which localizes a few amino acids before the C-terminal hydrophobic domain. Interestingly, one of the intriguing features of parasite GPIs is that they could be released or shed from the cell surface of parasites. It has been reported that when parasites invade erythrocyte, the merozoite surface coat will be shed or left outside the erythrocyte (Nathaly Wieser et al., 2019) and the secreted proteins were soluble and different from those on the surface coat of merozoites (Casanova et al., 2006). These reported features are not completely in agreement with the findings in the present study. Nathaly Wieser et al. (2019) mentioned that parasite proliferation was not negatively affected by antibodies against the soluble protein *in vivo*, so that the parasites exposed to the immune system could achieve the immune evasion, but this assumption requires to be confirmed with further tests.

In the present study, BmSA1 was identified with abundant expression in both the membrane and cytoplasm of the parasites, as shown by indirect immunofluorescence assay. Furthermore, the diffuse distribution of BmSA1 outside the parasites could be observed from the three-dimensional perspective under a laser scanning confocal microscope, which directly confirms that BmSA1 can be secreted outside the parasites.

The MSPs of parasites are known to play a role in the initial phase of erythrocyte invasion through the interaction of the merozoite with the host cell, and it is the same for GPIs. To further identify the function of BmSA1, RBC-binding assays were performed and rBmSA1-GST was found able to adhere to RBCs. Additionally, the amount of adhesion is proportional to that of protein, indicating that the adhesion is independent of receptor-ligand binding (Rodriguez et al., 2008). Previous work suggested that anti-SAG1 antiserum could inhibit parasite invasion by blocking native SAG1 on the cell surface of *T. gondii* (Mineo et al., 1993). In this study, we performed an antiserum neutralization test in *in vitro* culture and found that anti-BmSA1 antisera could inhibit the growth of parasites, with the highest inhibition rate observed at 240 mg/mL. Nonetheless, previous studies have shown a significant difference between *in vitro* culture and *in vivo* environment. For example, it has been reported that the antiserum raised against BmAMA1 can effectively block the invasion of *B. microti* into host cells in an *in vitro* culture environment (Moitra et al., 2015), but the mice immunized with rBmAMA1 did not show any significant protection after *B. microti* challenge (Wang et al., 2017). Therefore, more evidence needs to be gathered to support that BmSA1 is important for the invasion of *B. microti* into host cells in an *in vivo* environment.

In this study, we demonstrated that BmSA1 exists in parasites in two forms and is located on both the membrane and cytoplasm. Moreover, RBC binding assays showed that BmSA1 plays an important role in binding host RBCs. The antiserum neutralization test suggested that anti-BmSA1 antiserum can inhibit merozoite invasion into the erythrocytes of parasites. These results suggest the potential of BmSA1 as a vaccine candidate. Further studies should focus on whether BmSA1 is still a crucial virulence factor in the *in vivo* environment.

## Data Availability Statement

The datasets generated for this study are available on request to the corresponding author.

## Ethics Statement

The animal study was reviewed and approved by the Laboratory Animals Research Centre of Hubei Province and Huazhong Agricultural University.

## Author Contributions

All authors contributed to this work. ML, YA, JG, ZN, QL, XL, LH, and JZ conceived and designed the project. ML, YA, XZ, SW, YZ, and XA performed the experiments. ML, JG, YA, and ZN analyzed the data. ML and LH wrote and edited the manuscript.

## Conflict of Interest

The authors declare that the research was conducted in the absence of any commercial or financial relationships that could be construed as a potential conflict of interest.

## Footnotes

1 http://gpcr.biocomp.unibo.it/predgpi/pred.htm

2 http://www.cbs.dtu.dk/services/SignalP-4.1
